# Lateral opening of the bacterial translocon on ribosome binding and signal peptide insertion

**DOI:** 10.1038/ncomms6263

**Published:** 2014-10-15

**Authors:** Yan Ge, Albena Draycheva, Thomas Bornemann, Marina V. Rodnina, Wolfgang Wintermeyer

**Affiliations:** 1 Department of Physical Biochemistry, Max-Planck-Institute for Biophysical Chemistry, 37077 Göttingen, Germany

## Abstract

Proteins are co-translationally inserted into the bacterial plasma membrane via the SecYEG translocon by lateral release of hydrophobic transmembrane segments into the phospholipid bilayer. The trigger for lateral opening of the translocon is not known. Here we monitor lateral opening by photo-induced electron transfer (PET) between two fluorophores attached to the two SecY helices at the rim of the gate. In the resting translocon, the fluorescence is quenched, consistent with a closed conformation. Ribosome binding to the translocon diminishes PET quenching, indicating opening of the gate. The effect is larger with ribosomes exposing hydrophobic transmembrane segments and vanishes at low temperature. We propose a temperature-dependent dynamic equilibrium between closed and open conformations of the translocon that is shifted towards partially and fully open by ribosome binding and insertion of a hydrophobic peptide, respectively. The combined effects of ribosome and peptide binding allow for co-translational membrane insertion of successive transmembrane segments.

## Abstract (web summary)

Integral membrane proteins laterally partition from the SecYEG translocon into the phospholipid bilayer. Here, the authors use photo-induced electron transfer to show that ribosome binding induces the opening of the lateral gate, and demonstrate that lateral opening does not happen at low temperature.

## Graphical abstract

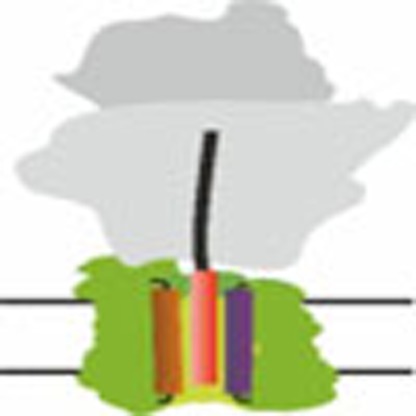

Integral membrane proteins are inserted into the bacterial cytoplasmic membrane in a co-translational manner. Ribosomes that are synthesizing membrane proteins are targeted to the membrane by the signal recognition particle (SRP) pathway. SRP recognizes those ribosomes by binding to a so-called signal-anchor sequence (SAS) that forms the first transmembrane (TM) segment of the membrane protein. The translating ribosome is targeted to the protein-conducting pore in the membrane, the translocon, that ensures the sequential membrane insertion of TM segments together with the necessary topological arrangements of TM segments and the connecting intra- or extracellular loops.

The bacterial translocon consists of a ternary complex of proteins SecY, SecE and SecG (SecYEG). The crystal structure of the homologous SecYEβ from *Methanococcus jannaschii*^1^ as well as crystal structures of bacterial SecYEG^2^,^3^,^4^ shows the translocon as a pseudosymmetrical structure with TM segments 1–5 and 6–10 of SecY forming a central pore that in the resting state is closed by a small plug helix. The pore can open in two ways^5^. For protein translocation through the translocon into the periplasm, the plug domain closing the pore towards the periplasm is moved to the side. Alternatively, to allow for TM segments of membrane proteins to enter the lipid phase, the two halves of the translocon move apart and open laterally. Once the lateral gate is opened, TM segments of nascent peptides can partition between the hydrophilic inner pore of the translocon and the hydrophobic lipid phase according to their hydrophobicity^6^,^7^. The exit of peptides into the lipid phase may also be controlled kinetically, in that the hydrophobicity of the peptide determines lateral gate opening^8^. A gating motif of conserved amino acids has been identified that is located between the two translocon halves and sets the hydrophobicity threshold for peptides to move into the phospholipid bilayer^9^.

A partially open lateral gate of SecY was revealed by the crystal structure of SecYEβ from *Pyrococcus furiosus*^3^, and opening of the gate was attributed to the binding of the C-terminal peptide of one SecY to a neighbouring SecYEβ in the crystal. A slight movement of the two SecY helices forming the gate towards open as induced by binding an oligopeptide mimicking a preprotein was also observed in two-dimensional crystals of SecYEG^10^. While these data suggested an active role of peptide insertion in lateral channel opening, the structure of the SecYEG–SecA complex revealed a partially open lateral window as well, indicating that binding of a cytosolic ligand—SecA in that case—to the cytosolic loops of SecY can induce a change at the lateral gate^4^. Lateral gate opening induced by SecA binding was also shown by single-molecule fluorescence measurements with nanodisc-embedded SecYEG^11^. In fact, opening of the gate appears to be coupled to protein translocation through the translocon, as preventing opening by crosslinking the two helices forming the rim of the gate impaired translocation^12^. Cryo-EM reconstructions of ribosome–translocon complexes show the translocon with a hydrophobic nascent peptide outside the partially opened gate^13^,^14^,^15^; a similar structure was observed with an ribosome-nascent-chain complex (RNC)–translocon complex with two TM segments^16^. For these complexes it is not clear when and by which ligand—ribosome or hydrophobic peptide—opening of the gate was triggered, although in the SecYEG complexes with non-translating ribosomes from either *M. jannaschii* or *Escherichia coli* the lateral gate appeared to be closed^14^. Molecular dynamic simulations suggested conformational changes or partial opening of the lateral gate on binding of a ribosome to the cytosolic loops of SecY^17^,^18^,^19^ or the insertion of a hydrophobic peptide in the channel^20^.

Here, we set out to examine lateral gating of the translocon by using *E. coli* SecYEG integrated into nanodiscs^21^ and employing a fluorescence approach, which is photo-induced electron transfer (PET) between two labels inserted into the translocon. PET takes place at very short distances between a fluorophore and a quenching group^22^. Therefore, PET appears to be ideally suited for the short distance between the two helices of SecY (7 and 2b) forming the rim of the lateral gate, about 7 Å (ref. 1), and the small distance change expected for opening the lateral gate, to about 24 Å (ref. 13). A fluorophore, BODIPY FL, and a quencher, tryptophan, that formed a suitable PET pair were incorporated into SecY helices 7 and 2b. We monitored the extent of PET in the complexes of the double-labelled translocon with either vacant *E. coli* ribosomes or RNCs exposing nascent peptide chains of varying hydrophobicity. The results suggest that ribosome binding alone enhances the separation of the two helices. The effect is reinforced with ribosomes exposing hydrophobic TM segments, suggesting that ribosome binding and hydrophobic peptide interactions within the translocon pore both contribute to lateral gate opening. We also observe that gate opening strongly depends on the temperature, in that the closed conformation of the translocon prevails at low temperature, even in the complex with ribosomes.

## Results and Discussion

### Labelling the translocon

**Figure 1:**
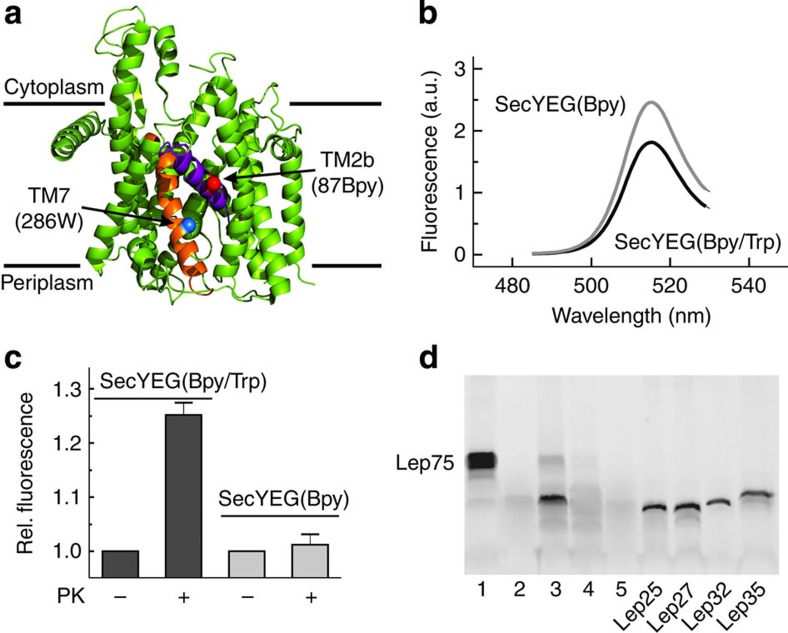
PET between fluorophores at the rim of the lateral gate of the translocon. (**a**) Structure of SecYEβ from *M. jannaschii*^1^. TM segments 2b (purple) and 7 (orange) with positions 87 and 286 for Bpy labelling (red sphere) and Trp insertion (blue sphere), respectively, are indicated. The other parts of SecYEβ are coloured green. (**b**) Quenching of the fluorescence of Bpy in SecYEG(Bpy/Trp) inserted in nanodiscs. The emission spectra of solutions of SecYEG(Bpy) (light grey) and SecYEG(Bpy/Trp) (dark grey) of equal concentrations (by absorbance at 205 nm) were measured on excitation at 475 nm and are plotted in arbitrary units (a.u.) (Methods). The signal of the buffer control was practically negligible in the peak area and was subtracted. (**c**) Effect on Bpy fluorescence of proteinase K digestion of double- and single-labelled translocon. SecYEG(Bpy/Trp) and SecYEG(Bpy), both embedded in nanodiscs, were digested with proteinase K (Methods), monitoring Bpy fluorescence at 25 °C. Data are represented as mean±s.e.m. (*N*=3) and are plotted as the ratio relative to the respective signal measured before digestion set to 1.0. (**d**) Protection against proteinase digestion of the N-terminal SAS of Lep75-RNC bound to the translocon. Bpy-Lep75-RNC carrying a Bpy label at the N-terminal methionine (1) was digested with proteinase K in the absence of translocon (2), in the presence of nanodisc-embedded translocon (3), in the presence of empty nanodiscs (4) and in the presence of bovine serum albumin in an amount equivalent to that added by the nanodisc-embedded translocon (5) (Methods). Marker peptides were prepared from Bpy-Lep-RNCs carrying Bpy-labelled nascent peptides of the indicated lengths.

The bacterial SecYEG translocon consists of a ternary complex of SecY, SecE and SecG. In the crystal structure of the homologous SecYEβ from *M. jannaschii* TM helices 7 and 2b of SecY are located close to one another and form the rim of the lateral gate^1^, suggesting the two helices are suitable for the insertion of a PET couple to report on gate opening. For fluorophore insertion into helices 7 and 2b of *E. coli* SecYEG, we chose residues F286 (helix 7) and S87 (helix 2b), based on the reported disulfide bridge formation between Cys residues inserted into those two positions^12^ (Fig. 1a). We replaced residue S87 with Cys and reacted the substituted SecYEG with the maleimide derivative of the fluorescent boron-dipyrromethene dye BODIPY FL (Bpy) to obtain single-labelled SecYEG(Bpy) (Methods). To introduce a PET quencher, F286 in helix 7 was replaced with Trp, yielding double-labelled SecYEG(Bpy/Trp). Measurements with model compounds have shown that Trp strongly quenches the fluorescence of Bpy^23^. In fact, Trp insertion into Bpy-labelled SecYEG resulted in quenching of the Bpy fluorescence (Fig. 1b), indicating that Bpy and Trp in double-labelled SecYEG were engaged in PET, as expected.

The extent of PET quenching was determined quantitatively by proteinase K digestion of double-labelled SecYEG(Bpy/Trp) inserted into nanodiscs (see the following paragraph). On complete digestion, the Bpy fluorescence of SecYEG(Bpy/Trp) increased by about 25%, whereas no fluorescence change was observed on proteinase digestion of single-labelled SecYEG(Bpy) (Fig. 1c). These observations indicate that in the intact complex Bpy and Trp undergo PET, resulting in Bpy fluorescence quenching, and that PET is suppressed on separation of the fluorophores by proteinase digestion of the double-labelled translocon.

### Incorporation of the translocon into nanodiscs

As a membrane protein complex, SecYEG exposes hydrophobic residues at its outside, making it aggregation-prone in aqueous solution. Aggregation can be avoided by adding detergent or by incorporating the translocon into phospholipid bilayers, as in proteoliposomes or nanodiscs. Nanodiscs, discoidal lipid bilayers encircled by a membrane scaffold protein derived from apolipoprotein A1, have proven to be effective in solubilizing membrane proteins and have been used to study a wide variety of purified membrane proteins^24^.

Monomers of SecYEG reconstituted into nanodiscs were previously shown to be active in pre-protein translocation^11^. We examined the functional integrity of the nanodisc-embedded SecYEG translocon by forming complexes with RNCs that exposed nascent chains of membrane or non-membrane proteins and assaying for protection against proteinase K digestion. As RNC presenting a nascent membrane protein we used Lep75-RNC, carrying 75 N-terminal amino acids of leader peptidase (Lep) of which about 40 residues were exposed outside the ribosomal peptide exit tunnel, including a hydrophobic SAS (Supplementary Fig. 1a). To visualize the N-terminal peptide of leader peptidase in the digestion experiments, the nascent peptide was labelled with Bpy at the N-terminal Met residue^25^.

On limited proteinase K digestion of the translocon−Lep75-RNC complex, an N-terminal fragment of a length of about 30 amino acids was protected (Fig. 1d). A protected fragment of that size would be expected when the hydrophobic signal sequence was inserted into the translocon or had moved into the lipid bilayer. No protected fragment was observed with empty nanodiscs and Lep75-RNC. We also assayed HemK75-RNC, which does not contain a hydrophobic signal sequence, by proteinase K digestion and did not observe peptide protection by the translocon (Supplementary Fig. 2a), although a complex was formed, as shown below. Protection of the nascent Lep signal peptide against proteinase K digestion was also observed with single- or double-labelled translocon (Supplementary Fig. 2b). Thus, introducing Bpy at position 87 and Trp at position 286 in SecY did not interfere with the function of the translocon in productive binding of Lep75-RNC.

### Ribosome binding promotes lateral opening of the translocon

**Figure 2:**
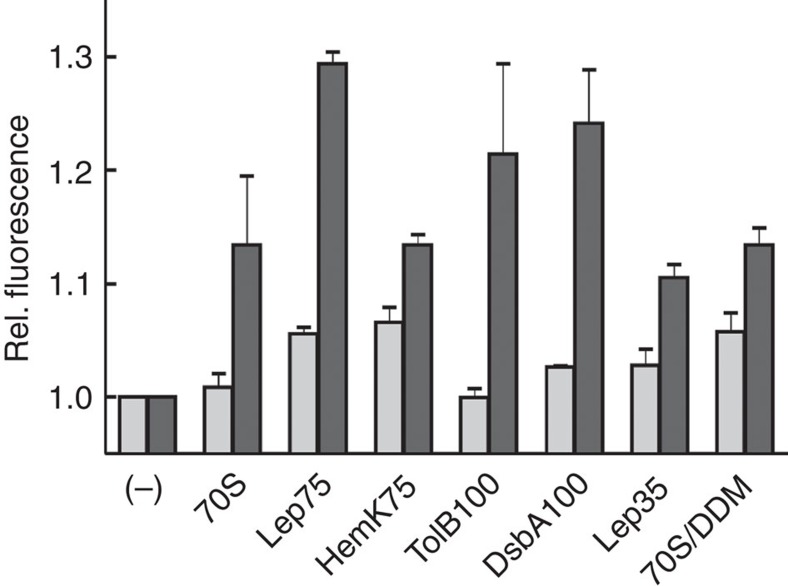
Lateral gate opening of the translocon by ribosome or RNC binding. Non-translating ribosomes (70S) or RNCs (200 nM) were added to nanodisc-embedded single-labelled SecYEG(Bpy) (light bars) or double-labelled SecYEG(Bpy/Trp) (dark bars) (50 nM), as indicated, and the change of Bpy fluorescence was measured (25 °C). 70S/DDM: in these samples, single- or double-labelled translocons were not embedded in nanodiscs, but solubilized by adding the detergent DDM (0.03%) (Methods). Data are represented as mean±s.e.m. (*N*=3) and plotted relative to the respective initial signal measured before the addition of ribosomes or RNCs (−).

Vacant 70S ribosomes and Lep75-RNCs bound the nanodisc-embedded translocon with high affinity, *K*_d_ values being around 20 and 10 nM, respectively (Supplementary Fig. 3), comparable to values reported previously^26^. To examine the effect of ribosome binding on the conformation of the lateral gate, we added a saturating amount of vacant ribosomes or RNCs to nanodisc-embedded SecYEG(Bpy/Trp), monitoring Bpy fluorescence; control measurements were performed with SecYEG(Bpy) (Fig. 2). On addition of vacant 70S ribosomes, the Bpy fluorescence signal of SecYEG(Bpy/Trp) increased by 15%, whereas with SecYEG(Bpy) no fluorescence change was observed. A much larger effect, close to 30% signal increase, was brought about by adding Lep75-RNC to SecYEG(Bpy/Trp), whereas the effect was only about 5% with SecYEG(Bpy). Thus, the net effect of Lep75-RNC binding observed with double-labelled translocon, 25%, corresponds to the maximum PET effect determined by proteinase digestion (Fig. 1c).

Two other RNCs exposing N-terminal signal sequences of somewhat lower hydrophobicity, TolB100-RNC and DsbA100-RNC (Supplementary Fig. 1), increased the signal slightly >20%. HemK75-RNC, exposing a hydrophilic nascent chain, had a similar effect as non-translating ribosomes. The same moderate effect was observed with Lep35-RNC, indicating similar effects of translocon binding to vacant ribosomes, to RNCs presenting a hydrophilic nascent chain and to RNCs carrying a nascent peptide within the exit tunnel. With SecYEG(Bpy/Trp) solubilized by adding detergent (DDM) and non-translating ribosomes, we observed a fluorescence increase by about 15%, as with SecYEG(Bpy/Trp) embedded in nanodiscs. The controls with single-labelled SecYEG(Bpy) showed signal changes of 5% or less (Fig. 2).

These results indicate that SecY helices 7 and 2b move apart, or are stabilized in the open conformation, when a ribosome binds to the cytosolic side of the translocon and that the effect is reinforced when a hydrophobic TM segment, such as the SAS of leader peptidase or the signal sequences of TolB or DsbA, enters the translocon pore or passes through the lateral gate to reach the phospholipid bilayer. Nascent peptides of varying hydrophobicity elicited somewhat different effects, in keeping with the proposal of a gating motif in the translocon that sets the hydrophobicity threshold for peptides to move into the phospholipid bilayer^9^. The efficiency of PET between Bpy in helix 2b and Trp in helix 7 of SecY presumably decreases because the lateral gate opens further to allow for the passage of the peptide.

### Isolated signal peptide does not trigger gate opening

**Figure 3:**
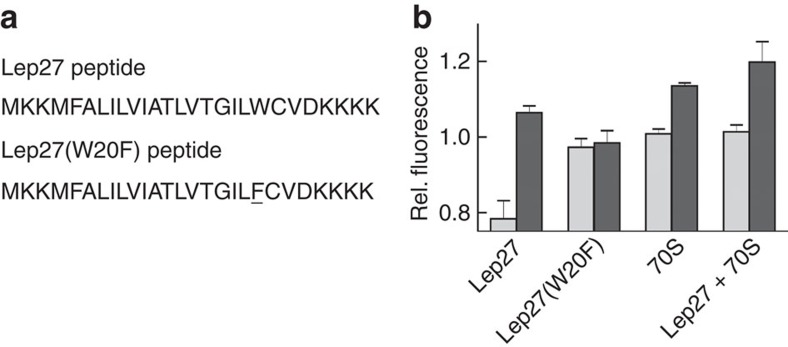
Effect of the isolated Lep27 peptide on lateral translocon gating. (**a**) Sequences of Lep27 peptides. The SAS of Lep comprises residues 4 to 22. In Lep(W20F), the Phe residue replacing Trp is underlined. (**b**) Effect of Lep27 peptides. The Bpy fluorescence of SecYEG(Bpy) (light bars) or SecYEG(Bpy/Trp) (dark bars) was measured on addition of the Lep27 peptide, the Lep27 peptide plus vacant ribosomes or the Lep27(W20F) peptide. For comparison, the effect of adding 70S ribosomes alone (Fig. 4) is also shown. Fluorescence intensities are plotted relative to the intensity (set to 1.0) measured before the addition of peptide and/or ribosomes. Data are represented as mean±s.e.m. (*N*=3).

We have also examined whether lateral gate opening is elicited by an isolated hydrophobic peptide of 27 amino acids encompassing the SAS of Lep (Lep27 peptide; Fig. 3a). The Lep27 peptide strongly quenched the Bpy fluorescence of SecYEG(Bpy), whereas the fluorescence of SecYEG(Bpy/Trp) was not changed much (Fig. 3b). The quenching could be caused by a Trp residue contained in the Lep peptide (position 20) that may come close to the Bpy residue attached to SecY and quench its fluorescence by PET. In fact, when we exchanged Trp20 with Phe to yield Lep27(W20F), the quenching effect with SecYEG(Bpy) was eliminated, whereas the fluorescence of SecYEG(Bpy/Trp) did not change significantly on addition of Lep27(W20F). Finally, the effect of vacant ribosomes and Lep27 peptide together was comparable to the signal increase induced by vacant ribosomes alone (Fig. 3b). The fluorescence effect induced by Lep75-RNC binding to double-labelled translocon was similar to Lep75(W20F)-RNC (Supplementary Fig. 4), indicating that the Trp residue in the nascent peptide of Lep75-RNC did not influence the Bpy label attached to helix 2b of SecY. Thus, according to these data, the Lep27 peptide without the RNC context did not seem to promote gate opening beyond that induced by ribosome binding alone. However, the data do not exclude a transient effect of the peptide. A possible scenario would be that the Lep peptide initially induced opening of the gate, but subsequently moved into the surrounding phospholipid bilayer, allowing the translocon to close again.

### Lateral translocon opening is blocked at low temperature

**Figure 4:**
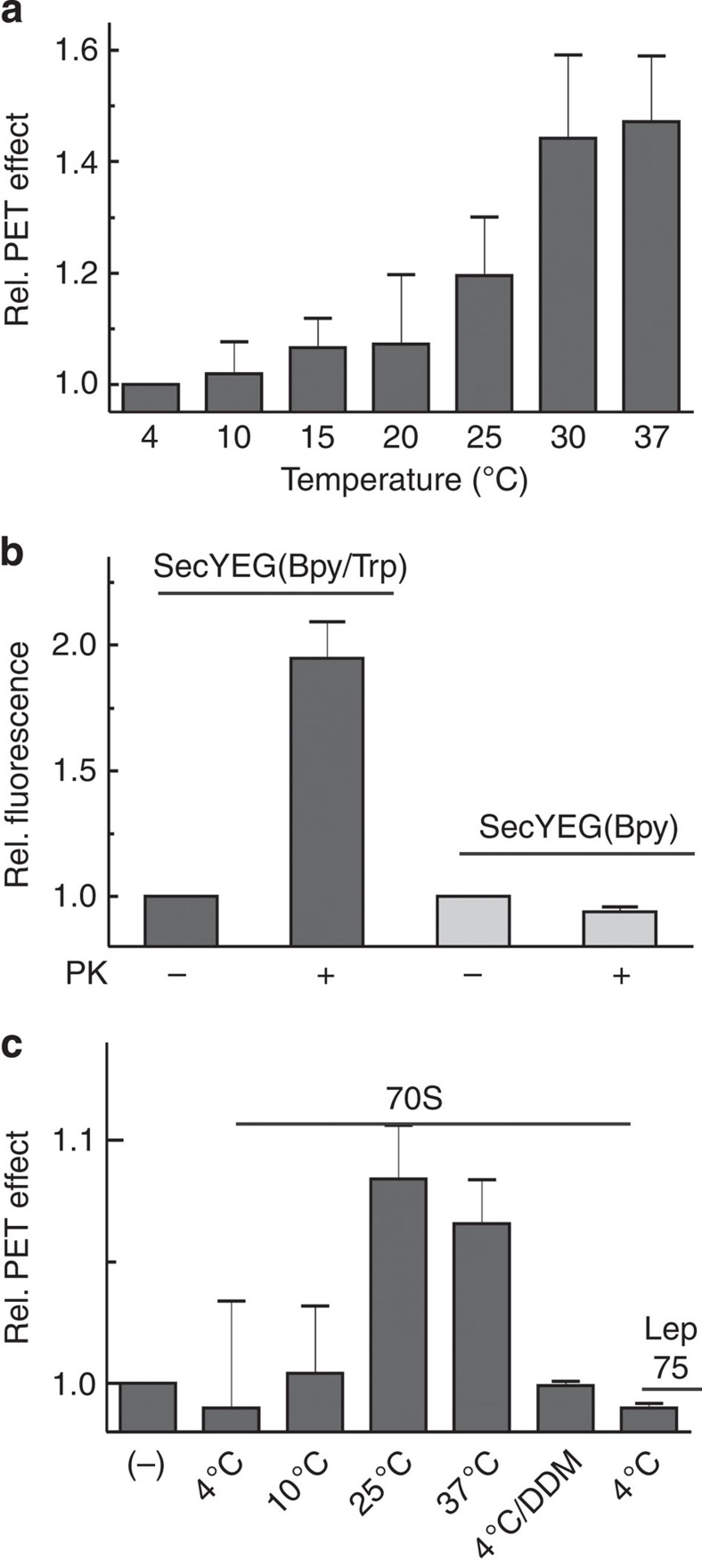
Temperature dependence of lateral translocon opening. (**a**) Influence of temperature on spontaneous translocon opening. The fluorescence of single-labelled SecYEG(Bpy) and double-labelled SecYEG(Bpy/Trp) was measured at different temperatures. Fluorescence values measured for the double-labelled translocon are normalized by the respective values measured for the single-labelled translocon and plotted as PET effect relative to the value obtained at 4 °C set to 1.0. Data are represented as mean±s.e.m. (*N*=3). (**b**) Proteinase K digestion of double-labelled translocon at low temperature. The fluorescence increase accompanying proteinase K digestion of SecYEG(Bpy/Trp) (dark grey), measured at 4 °C, is shown along with the control digestion of single-labelled SecYEG(Bpy) (light grey). Data are presented as mean±s.e.m. (*N*=3) and plotted relative to the respective initial signal before digestion. (**c**) Influence of temperature on ribosome-induced gate opening. The effect of binding vacant 70S ribosomes or Lep75-RNCs to labelled translocons was measured as in Fig. 2, except that the temperature was varied as indicated. Fluorescence values for double-labelled translocon are normalized by the signal changes of the controls with single-labelled translocon and plotted relative to the fluorescence measured at the respective temperature before the addition of ribosomes (−). The effect of 70S ribosome binding at 4 °C to the translocon solubilized by adding DDM is shown for comparison. Data are represented as mean±s.e.m. (*N*=3).

In a recent cryo-EM reconstruction of a SecYEG translocon bound to vacant ribosomes the lateral gate appeared to be closed^14^. As the complex for the cryo-EM analysis was prepared at low temperature (on ice), we have examined whether lateral gating in our system was influenced by the temperature. First, we measured the temperature dependence of the fluorescence of single- and double-labelled translocons. As expected, the Bpy fluorescence decreased with increasing temperature in both cases, but the effect was much smaller for the double-labelled translocon, resulting in a net fluorescence increase at increasing temperature (Fig. 4a). This indicates that the quenching caused by PET was suppressed with increasing temperature, suggesting that lateral gate opening was enhanced up to a temperature of 30 °C.

It is known that the *E. coli* plasma membrane undergoes a phase transition in the temperature range between 10 and 30 °C (ref. 27), which could also influence the fluorescence properties of the Bpy label. However, such an effect probably does not contribute much to the fluorescence change observed here, as we observed a similarly small effect of ribosome binding on the fluorescence of double-labelled translocon solubilized with DDM (Fig. 4c), indicating that the closed translocon conformation was stabilized at low temperature. This conclusion is supported by the results of proteinase digestion. The fluorescence increase resulting from proteinase K digestion of double-labelled translocon, which was about 25% at 25 °C (Fig. 1c), was much larger, about 100%, at 4 °C, whereas the control performed with single-labelled translocon showed no difference (Fig. 4b). As the fluorescence level after digestion is the same for single- and double-labelled translocon, the difference observed with the double-labelled translocon indicates that lowering the temperature strongly favoured the closed conformation of the translocon.

The temperature effect may suggest that the translocon undergoes thermally driven fluctuations between closed and (partially) open conformations. The effects of binding 70S ribosomes or Lep75-RNCs to SecYEG that we observed at 25 °C (Fig. 1c) vanished at 4 °C (Fig. 4c), as the fluorescence change induced by ribosome binding was the same for single- and double-labelled SecYEG. Apparently, at the lower temperature the closed form of the translocon was stabilized such that ribosome binding to the translocon did not induce or stabilize the open conformation of the lateral gate. That the complex was formed at the lower temperature at even slightly higher affinity, *K*_d_=10 nM compared with 20 nM, was confirmed by titrations monitoring FRET (fluorescence resonance energy transfer) between labels in the translocon and the ribosome (Supplementary Fig. 3c).

## Conclusions

**Figure 5:**
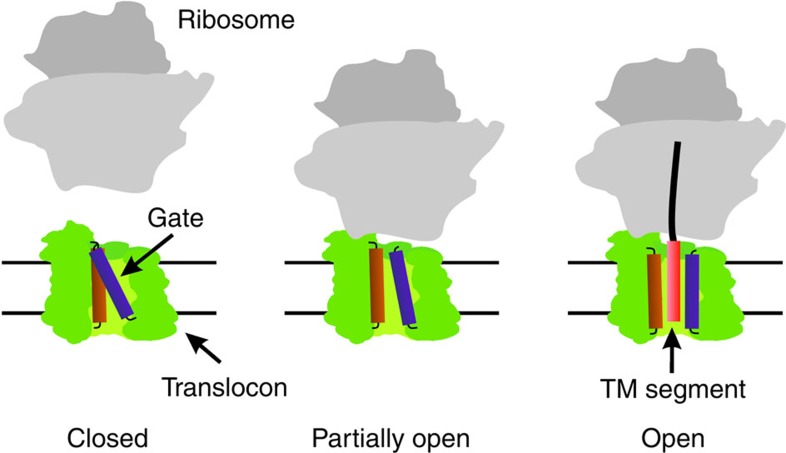
Stepwise lateral opening of the translocon by ribosome binding and hydrophobic peptide insertion. The translocon (green) with the two transmembrane segments of SecY, TM7 (orange) and TM2b (purple) that close the lateral gate is shown in the closed state (left), partially open (middle) with a bound ribosome (grey) or fully open (right) with a bound RNC inserting a hydrophobic TM segment (red) into the central pore. The partially open state can also be induced by a ribosome exposing a non-hydrophobic nascent chain (not depicted).

The present results indicate that translocon binding to vacant ribosomes induces partial opening of the lateral gate. The effect of vacant ribosomes is reinforced with translating ribosomes exposing hydrophobic signal sequences, suggesting that ribosome binding to the cytosolic loops of SecY and peptide insertion into the translocon work together to induce the fully open conformation (Fig. 5). In contrast, a peptide encompassing a TM segment added *in trans* did not stabilize the open conformation of the translocon, indicating that the RNC context is required to elicit the fully open conformation. Lateral gating is strongly dependent on temperature, in that the closed conformation is stabilized at low temperature to the extent that partial opening by ribosome binding does not take place. The translocon appears as a dynamic structure whose conformation changes between closed and open lateral gate and is modulated by interactions with various ligands, including ribosomes, SecA, and hydrophobic segments of nascent peptides exposed on translating ribosomes.

## Methods

### Materials

Experiments were performed in buffer A (20 mM HEPES, pH 7.5, 70 mM NH_4_Cl, 30 mM KCl, 7 mM MgCl_2_, 10% glycerol) at 25 °C, if not indicated otherwise. Ribosomes from *E. coli* MRE600, IF1, IF2, IF3, EF-Tu, EF-G, and f[^3^H]Met-tRNA^fMet^ were prepared according to standard protocols^28^. Bpy-[^3^H]Met-tRNA^fMet^ carrying Bpy at the alpha amino group of methionine was prepared^25^,^29^ by incubating [^3^H]Met-tRNA^fMet^ (30 μM) with BODIPY-FL sulfosuccinimidyl ester (Invitrogen) (4 mM) in buffer A (pH 8.5) for 4 min at 0 °C. The reaction was stopped by adding potassium acetate (pH 5) to 0.2 M and ethanol precipitation. Excess dye was removed by four additional precipitation steps. The resulting tRNA pellet was dried, dissolved in H_2_O and stored at −80 °C. The extent of Bpy modification was >80%, as assessed photometrically using extinction coefficients of 75 mM^−1^ cm^−1^ (505 nm) for Bpy and 575 mM^−1^ cm^−1^ (260 nm) for the tRNA.

### Expression and purification of SecYEG and MSP1D1

SecYEG containing N-terminally His6-tagged SecE was expressed from plasmid pTRC99a, which was kindly provided by Christiane Schaffitzel (EMBL, Grenoble Outstation) in Lemo21(DE3) cells (New England Biolabs) and purified following a published protocol^30^ with modifications. All steps were performed at 4 °C. Cells were opened using an Emulsiflex homogenizer in buffer B (20 mM Tris–HCl, pH 7.5, 200 mM NaCl, 5 mM MgCl_2_). Following centrifugation (30,000 *g*, 10 min), membranes were collected by ultracentrifugation (150,000 *g* for 120 min; Ti 50.2, Beckman Coulter), and extracted for 1 h in buffer C (20 mM Tris-HCl, pH 7.5, 1.0 M NaCl, 5 mM MgCl_2_, 5 mM imidazole, 0.5 mM phenylmethylsulfonyl fluoride (PMSF), 1 mM dithiothreitol (DTT), and 1% dodecyl-β-D-maltopyranoside (DDM)). The suspension was clarified by ultracentrifugation (75,000 *g* for 25 min; Ti 50.2 rotor; Beckman Coulter). Protein in the supernatant was subjected to affinity chromatography on Ni-NTA agarose. Protein was loaded onto the column, washed with buffer D (20 mM Tris, pH 7.5, 200 mM NaCl, 5 mM MgCl_2_, 10% glycerol, 0.03% DDM, 10 mM imidazole) and eluted with buffer D supplemented with 200 mM imidazole. Fractions containing SecYEG were dialysed against buffer E (50 mM HEPES, pH 8.0, 50 mM NaCl, 10% glycerol, 0.03% DDM). SecYEG was further purified by cation exchange chromatography on SP-Sepharose Fast Flow resin (GE Healthcare) in buffer E, using a gradient of 50–600 mM NaCl. Eluted SecYEG was concentrated and re-buffered into buffer A supplemented with 0.03% DDM. Concentrations of SecYEG were determined by absorbance at 205 nm.

SecY mutants SecY(F286W) and SecY(S87C) were generated using the QuikChange PCR mutagenesis and expressed and purified as wild-type SecY. The membrane-scaffold protein MSP1D1 containing an N-terminal His7-tag was expressed from plasmid 20061 (Addgene) and purified by chromatography on Ni-NTA agarose^31^.

### Fluorescence labelling of SecYEG

SecYEG(S87C) or SecYEG(S87C/F286W) were labelled by incubation with a twofold molar excess of the maleimide derivative of BODIPY FL (Life Technologies) for 2 h at 25 °C in buffer F (20 mM HEPES, pH 7.0, 150 mM KCl, 10% glycerol, 0.03% DDM). The reaction was quenched by adding DTT (1 mM), and unreacted dye was removed from the labelled protein by gel filtration on Sephadex G25 (PD-10, GE Healthcare) in buffer A. The labelling efficiency was 90% for both SecYEG versions, as determined photometrically.

### Reconstitution of SecYEG into nanodiscs

For the assembly of nanodiscs containing SecYEG, we have modified a published protocol^21^,^32^. Purified scaffold protein MSP1D1 was dissolved in buffer F (20 mM HEPES, pH 7.0, 150 mM KCl, 10% glycerol, 0.03% DDM). Phospholipids from *E. coli* in chloroform solution (Avanti Polar Lipids) were dried under a stream of nitrogen. The lipids were resuspended in buffer A without glycerol and containing 0.5 M DDM. A typical reconstitution experiment involved mixing SecYEG, MSP1D1 and lipids at a molar ratio of 1:2:30. For the reconstitution of empty nanodiscs, the ratio of MSP1D1:lipids was increased to 1:60. After 1 h incubation on ice, self-assembly of nanodiscs was initiated by adding BioBeads (Bio-Rad) followed by gentle rocking overnight at 4 °C. Beads were removed by sedimentation, and the resulting mixture was centrifuged for 30 min at 15,000 r.p.m. and then injected onto a gel filtration column (Superdex 200 PG16/100) equilibrated with buffer A. Fractions containing nanodiscs were combined, concentrated (Vivaspin20, cut-off 100,000; Sartorius) and stored at −80 °C. If not stated otherwise, experiments were performed with SecYEG embedded in nanodiscs.

### RNC preparation

RNCs were prepared by *in vitro* translation of truncated mRNAs^33^, starting with purified 70S initiation complexes containing f[^3^H]Met-tRNA^fMet^ or Bpy-[^3^H]Met-tRNA^fMet^, as indicated. RNCs carrying nascent peptides used in this work were at least 80% pure.

### Proteinase K digestion of SecYEG and RNC-SecYEG complexes

To determine the maximum PET effect, single- or double-labelled translocons (50 nM) were completely digested with proteinase K (1.5 mg ml^−1^, 1 h, 37 °C); Bpy fluorescence was measured at 25 °C (Fig. 1c) or 4 °C (Fig. 4b) before and after digestion. To assess the protection of the N-terminal signal sequence by translocon binding, Bpy-Lep75-RNC (0.4 μM) was incubated with SecYEG (2.5 μM) for 5 min at 25 °C in a volume of 50 μl buffer A. Partial digestion with proteinase K in buffer A (1.5 mg ml^−1^) was performed for 1 h at 37 °C; samples were adjusted to 0.5 M NaOH and incubated for 30 min at 37 °C to inactivate proteinase K and liberate peptides from the tRNA. Samples (5 μl each) were loaded on Tricine-SDS–PAGE. N-terminal peptides were visualized by the fluorescence of Bpy (Fig. 1d). For assaying the ability of double-labelled SecYEG to protect the N-terminal peptide of Lep75-RNC, the RNC was prepared by *in vitro* translation, as above, using f[^3^H]Met-tRNA^fMet^ for initiation complex formation and visualizing the protected peptide by autoradiography (Supplementary Fig. 3).

### Fluorescence measurements

Fluorescence measurements were performed in buffer A on a Fluorolog-3 fluorimeter (Horiba) at 25 °C, unless indicated otherwise. Bpy emission was measured at 515 nm on excitation at 475 nm. Fluorescence signals were in the order of 200,000 c.p.s.

## Author contributions

Y.G., T.B., M.V.R. and W.W. designed experiments, Y.G. and A.D. performed experiments, Y.G. and T.B. analysed data, T.B. and W.W. wrote the manuscript.

## Additional information

**How to cite this article:** Ge, Y. *et al.* Lateral opening of the bacterial translocon on ribosome binding and signal peptide insertion. *Nat. Commun.* 5:5263 doi: 10.1038/ncomms6263 (2014).

## Supplementary Material

Supplementary Information Supplementary Figures 1-4 and Supplementary References
