# Supplementary material for: Lateral opening of the bacterial translocon on ribosome binding and signal peptide insertion
Source: Nat Commun. 2014 Oct 15;5:5263. doi: 10.1038/ncomms6263 (PMC4218953; doi:10.1038/ncomms6263)
Supplement: Supplementary Information — Supplementary Figures 1-4 and Supplementary References [file ncomms6263-s1.pdf]

Supplementary Information

Lateral opening of the bacterial translocon upon ribosome binding and signal peptide insertion

Yan Ge, Albena Draycheva, Thomas Bornemann, Marina V. Rodnina, Wolfgang Wintermeyer

Supplementary Figures

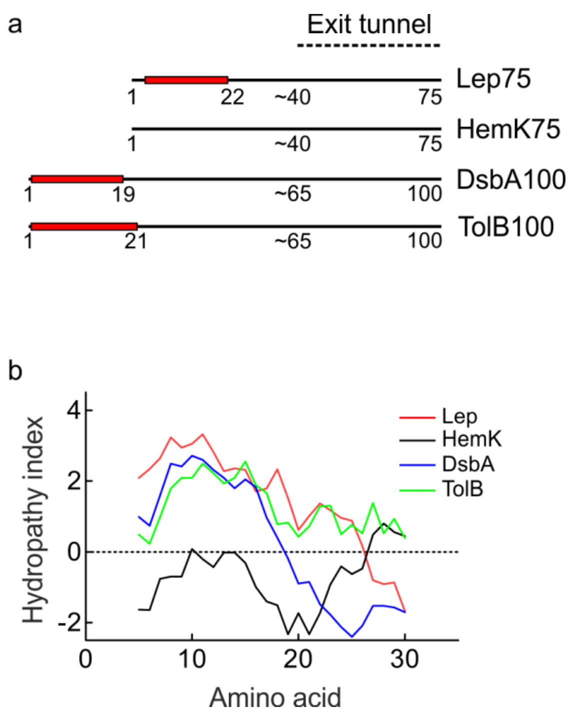

Supplementary Figure 1. RNC constructs used for translocon binding.

(**a**) RNCs prepared by translation of the respective truncated mRNAs. The length of the nascent peptides is indicated, as is the approximate length of the nascent peptide exposed outside the ribosome. This assumes that about 35 amino acids are contained within the exit tunnel, although this number may vary among constructs, depending on secondary structure formation within the tunnel. Signal sequences are depicted as red blocks.

(**b**) Hydropathy plots of the N-terminal sequences of RNCs. The plots were calculated as described [1].

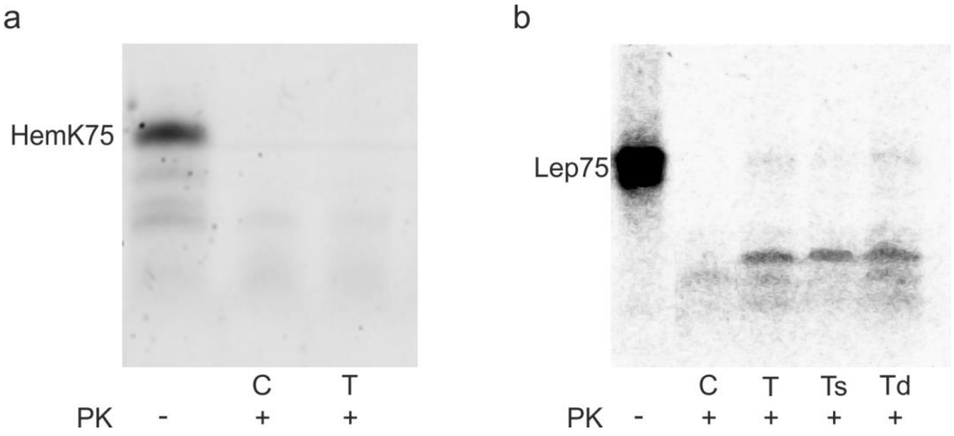

Supplementary Figure 2. Proteinase K digestion of RNCs and RNC-translocon complexes.

(**a**) HemK75-RNC. Bpy-labeled HemK75-RNC alone (C) and in complex with SecYEG-ND (T) was digested with proteinase K (Methods). HemK75 was visualized by the fluorescence of the N-terminal Bpy.

(**b**) Protection of Lep75-RNC bound to single- and double-labeled translocons. f[^{3}H]Lep75-RNC alone (C) or its complexes with unlabeled SecYEG (T), single-labeled SecYEG(Bpy) (Ts), and double-labeled SecYEG(Bpy/Trp) (Td) were treated with proteinase K as in **a**. Bands were visualized by autoradiography.

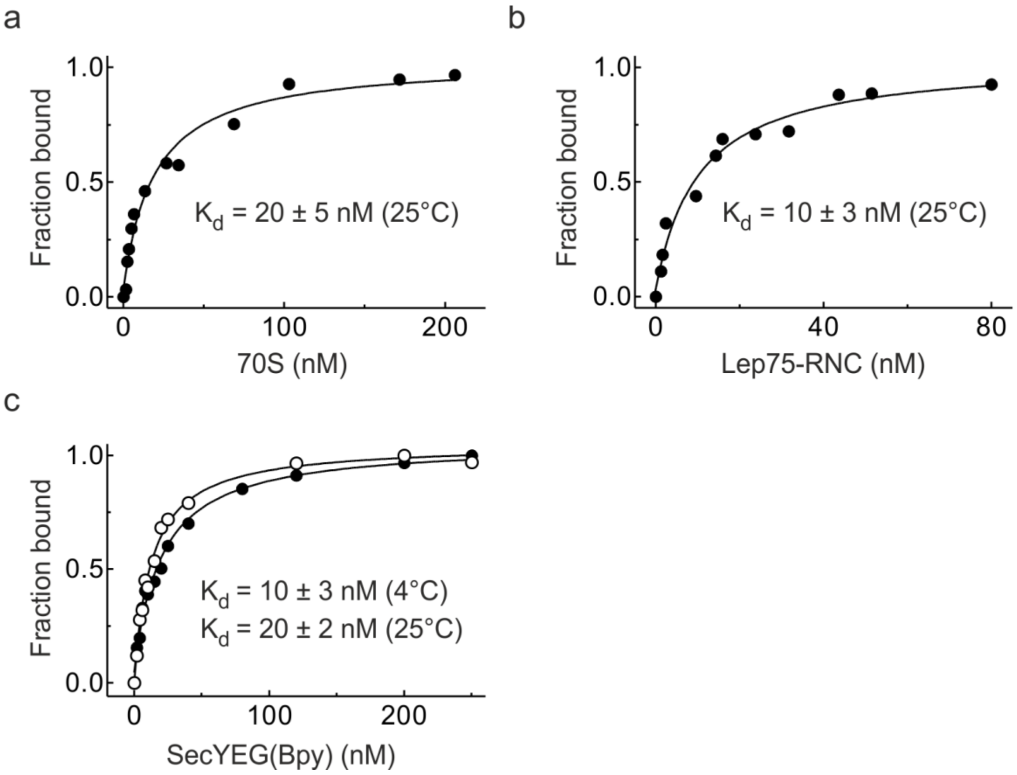

Supplementary Figure 3. Equilibrium titrations of SecYEG binding to ribosomes.

(**a**) Binding to non-translating 70S ribosomes. SecYEG(Bpy/Trp) (20 nM) was titrated with vacant ribosomes and the increase of Bpy fluorescence (about 25%) was measured, and normalized data are plotted. To obtain equilibrium dissociation constants (K_d), titrations were evaluated by non-linear fitting, using a quadratic equation that took into account the change of the ribosome concentration due to complex formation.

(**b**) Binding to Lep75-RNC. Titrations were performed and evaluated as in **a**, except that Lep75-RNC was used.

(**c**) Temperature effect on SecYEG binding to ribosomes. 70S ribosomes (5 nM) labeled at the ribosomal protein L23 with the FRET donor fluorophore MDCC [2] were titrated with nanodisc-embedded SecYEG(Bpy) labeled with the acceptor fluorophore Bpy at 4°C and 25°C. The decrease of donor fluorescence due to FRET (27% decrease) was monitored. Normalized data are plotted. The titrations were evaluated as in **a**.

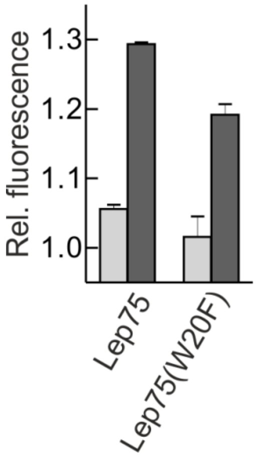

Supplementary Figure 4. PET effect of Lep75(W20F)-RNC.

The effect of adding Lep75(W20F)-RNC on the fluorescence of SecYEG(Bpy87/W286F) (dark bars) was measured by monitoring the fluorescence of Bpy. Control measurements were performed with single-labeled SecYEG(Bpy) (light bars). The data for Lep75-RNC (from Fig. 3) are included for comparison.

Supplementary References

1 Kyte, J. & Doolittle, R. F. A simple method for displaying the hydropathic character of a protein. *J Mol Biol* **157**, 105-132 (1982).

2 Bornemann, T., Holtkamp, W. & Wintermeyer, W. Interplay between trigger factor and other protein biogenesis factors on the ribosome. *Nat Commun* **5**, doi:10.1038/ncomms5180 (2014).
